# Tiliroside Attenuates NLRP3 Inflammasome Activation in Macrophages and Protects against Acute Lung Injury in Mice

**DOI:** 10.3390/molecules28227527

**Published:** 2023-11-10

**Authors:** Chao Zhong, Jing Yang, Keke Deng, Xiaoya Lang, Jiangtao Zhang, Min Li, Liang Qiu, Guoyue Zhong, Jun Yu

**Affiliations:** 1 Center for Translational Medicine, College of Traditional Chinese Medicine, Jiangxi University of Chinese Medicine, Nanchang 330004, China; 2 Center for Traditional Chinese Medicine Resources and Ethnic Medicine, Jiangxi University of Chinese Medicine, Nanchang 330004, China; 3 Department of Cardiovascular Sciences, Center for Metabolic Disease Research, Lewis Katz School of Medicine, Temple University, Philadelphia, PA 19140, USA

**Keywords:** tiliroside, NLRP3 inflammasome, macrophages, AMPK, mitochondrial damage, acute lung injury

## Abstract

The Nod-like receptor family PYRIN domain containing 3 (NLRP3) inflammasome is a multiprotein signaling complex that plays a pivotal role in innate immunity, and the dysregulated NLRP3 inflammasome activation is implicated in various diseases. Tiliroside is a natural flavonoid in multiple medicinal and dietary plants with known anti-inflammatory activities. However, its role in regulating NLRP3 inflammasome activation and NLRP3-related disease has not been evaluated. Herein, it was demonstrated that tiliroside is inhibitory in activating the NLRP3 inflammasome in macrophages. Mechanistically, tiliroside promotes AMP-activated protein kinase (AMPK) activation, thereby leading to ameliorated mitochondrial damage as evidenced by the reduction of mitochondrial reactive oxygen species (ROS) production and the improvement of mitochondrial membrane potential, which is accompanied by attenuated NLRP3 inflammasome activation in macrophages. Notably, tiliroside potently attenuated lipopolysaccharide (LPS)-induced acute lung injury in mice, which has been known to be NLRP3 inflammasome dependent. For the first time, this study identified that tiliroside is an NLRP3 inflammasome inhibitor and may represent a potential therapeutic agent for managing NLRP3-mediated inflammatory disease.

## 1. Introduction

Macrophage plays a pivotal role in the regulation of immune homeostasis by responding to pathogen- (PAMP) or damage- (DAMP) associated molecular patterns through pattern recognition receptors (PRRs) [1]. The NLRP3 inflammasome, which consists of an innate immune sensor Nod-like receptor (NLR) family PYRIN domain containing 3 (NLRP3), an adaptor apoptosis-associated speck-like protein containing CARD (ASC), and an effector protein Caspase-1, is a multiprotein complex that serves as an integral component of macrophage-related innate immunity [2]. Upon macrophage activation, NLRP3 recruits ASC and pro-Caspase-1, forming a molecular platform for autocatalytic activation of Caspase-1. The resulting active Caspase-1 then promotes the cleavage of pro-IL-1β and pro-IL-18 to generate biologically active pro-inflammatory cytokines IL-1β and IL-18, which are secreted into the extracellular milieu to induce inflammatory responses [3].

The activation of NLRP3 inflammasome is finely orchestrated by a two-step process, with “priming” mediated by the PRR/NF-κB signaling pathway that upregulates NLRP3 and pro-IL-1β expression and “assembly” mediated by PAMP- or DAMP-induced multiple pathways (including mitochondrial dysfunction, ion flux, and lysosomal disruption) leading to the formation of the NLRP3 inflammasome macromolecular complex [4]. The tightly regulated NLRP3 inflammasome has been recognized as beneficial in host defense against infections and cellular stresses [5,6]. However, mounting evidence demonstrates that the dysregulation of the NLRP3 inflammasome is implicated in the pathophysiology of various human diseases [7]. In acute inflammatory conditions like those in COVID-19, excessive production of IL-1β due to aberrant NLRP3 inflammasome activation triggers systemic hyperinflammation with a simultaneous “cytokine storm” characterized by the robust release of pro-inflammatory cytokines/chemokines, such as TNFA, IL-6, CXCL9, and CXCL10 [8,9]. These inflammatory responses eventually result in multiple organ dysfunction and even septic death [10]. Animal studies have shown that genetic deletion or pharmacological inhibition of the NLRP3 inflammasome protects against acute inflammatory insult in mice, manifesting lower systemic inflammation and alleviated organ damage with an improved survival rate [11,12]. In addition, the aberrant induction of NLRP3 inflammasome also contributes to the development of other complex diseases, including acute lung injury, atherosclerosis, type 2 diabetes, gout, lupus, rheumatoid arthritis, non-alcoholic steatohepatitis, and neurodegenerative disorders [13]. Given the involvement of the NLRP3 inflammasome in various diseases, it is not surprising that the NLRP3 inflammasome has been deemed as a promising target for the therapeutic intervention of NLRP3-related diseases [4,7].

Medicinal plants and bioactive natural products represent attractive sources for identifying agents against NLRP3 inflammasome activation [14,15]. Tiliroside, also known as kaempferol 3-*O*-glucoside-6″-E-coumaroyl, is a natural glycosidic flavonoid found in several medicinal and dietary plants [16]. Previous studies have shown that tiliroside has a wide range of pharmacological properties, such as anti-oxidant, immunomodulatory, anti-thrombotic, anti-aging, anti-cancer, and metabolic regulation effects [16]. Remarkably, tiliroside has been demonstrated to possess anti-inflammatory activity both *in vitro* and *in vivo*. Using lipopolysaccharide (LPS)-induced RAW264.7 macrophages, tiliroside treatment significantly reduced the production of IL-6, iNOS, COX-2, and NO through JNK and p38 MAPK inflammatory signaling pathways [17]. In an *in vitro* neuroinflammation model, tiliroside was found to target TRAF-6-mediated NF-κB and p38 MAPK signaling pathways to inhibit LPS-induced inflammatory activation of BV2 microglia [18], and its mode of action was dependent on the Nrf2 anti-oxidant mechanism [19]. Consistently, an *in vivo* study demonstrated that tiliroside exerts anti-inflammatory effects on phospholipase A2-induced mouse paw oedema and 12-*O*-tetradecanoylphorbol 13-acetate-induced mouse ear inflammation [20]. Furthermore, our recent study also showed that tiliroside attenuated intestinal inflammation and ulcerative colitis in mice by modulating M1/M2 macrophage polarization *in vivo* [21]. Although tiliroside has exhibited favorable pharmacological effects against inflammation, its influence on the activation of NLRP3 inflammasome and the underlying mechanism is still unclear. In this study, it was shown that tiliroside attenuates NLRP3 inflammasome activation in macrophages to exert its anti-inflammatory effect. By targeting the AMPK/mitochondrial damage pathway, tiliroside suppresses NLRP3 inflammasome assembly and the subsequent Caspase-1 activation and IL-1β production, leading to the amelioration of NLRP3-mediated inflammatory disease.

## 2. Results

### 2.1. Tiliroside Attenuates NLRP3 Inflammasome Activation in Macrophages

**Figure 1:**
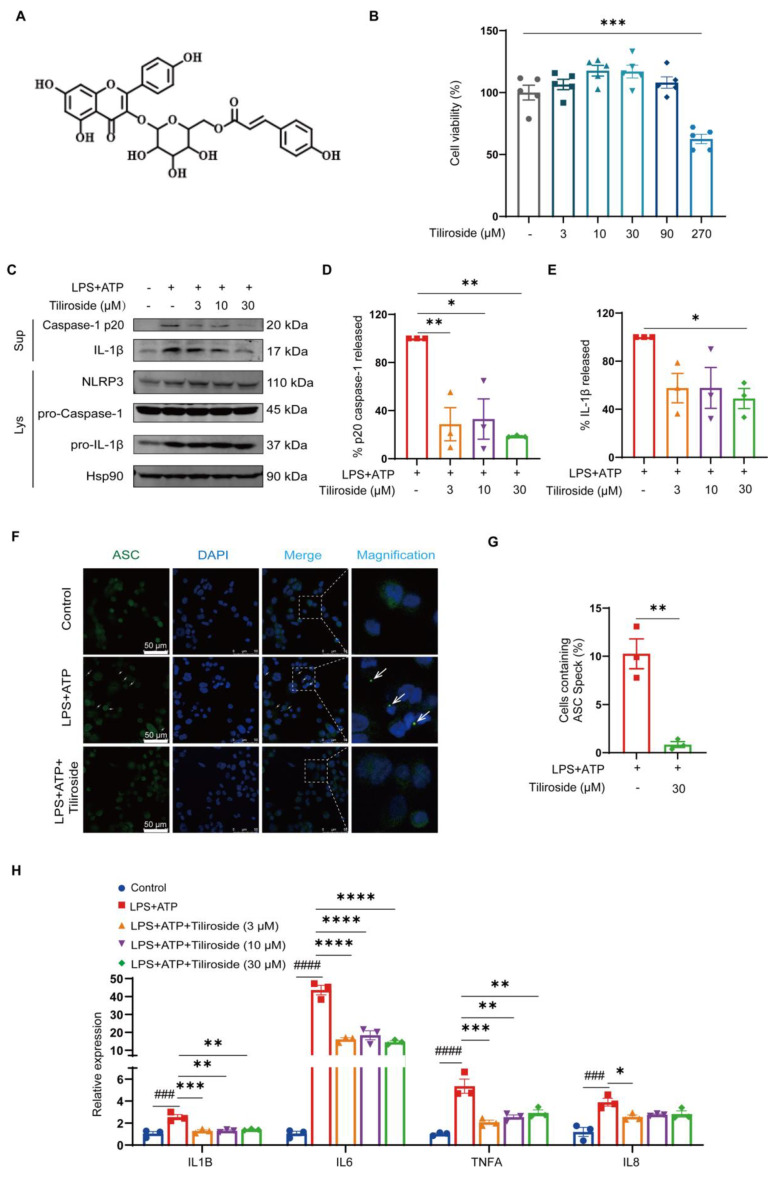
Tiliroside inhibits NLRP3 inflammasome activation in macrophages. (**A**) The chemical structure of tiliroside. (**B**) THP-1 macrophages were incubated with tiliroside (3, 10, 30, 90, 270 μM) for 30 h, and the cell viability was detected by MTT assay (n = 5). (**C**–**H**) THP-1 macrophages were pretreated with tiliroside or a vehicle for 24 h and then stimulated with LPS (2 μg/mL) for 5 h, followed by ATP (5 mM) treatment for 45 min. Protein levels of Caspase-1 p20 and IL-1β in culture supernatants (SN) and NLRP3, pro-Caspase-1, and pro-IL-1β in cell lysates (Lys) of THP-1 macrophages were determined by Western blotting (**C**). Relative Caspase-1 activation was shown as % of p20 release to the culture medium (n = 3) (**D**), and IL-1β maturation was shown as % of cleaved IL-1β release to the culture medium (n = 3) (**E**). Immunofluorescence staining detected ASC specks using anti-ASC (green) and counterstaining using DAPI (blue). The arrows indicate ASC specks (scale bar, 50 μm) (**F**). Quantification of the percentage of cells with ASC speck formation (n = 3) (**G**). IL-1β, IL-6, TNFA, and IL-8 gene expression levels were analyzed by qRT-PCR (n = 3) (**H**). Data are mean ± SEM. * *p* < 0.05, ** *p* < 0.01, *** *p* < 0.001, **** *p* < 0.0001, ^###^ *p* < 0.001, ^####^ *p* < 0.0001.

To investigate whether tiliroside (chemical structure shown in Figure 1A) affects NLRP3 inflammasome activation, THP-1-derived macrophages primed with LPS followed by adenosine triphosphate (ATP) stimulation were used as an *in vitro* model to activate the NLRP3 inflammasome. The effect of tiliroside on THP-1-derived macrophage viability was determined by using the MTT assay. Our result showed that tiliroside within the concentrations between 3–90 μM did not induce obvious cell cytotoxicity (Figure 1B). Next, THP-1-derived macrophages were pretreated with tiliroside (3, 10, 30 μM) or a vehicle for 24 h, and then stimulated with LPS (2 μg/mL) for 5 h followed by ATP (5 mM) for 45 min. It was noted that the maturation of pro-Caspase-1 to Caspase-1 p20 and the cleavage of pro-IL-1β to IL-1β were significantly induced by LPS plus ATP, suggesting the activation of the NLRP3 inflammasome (Figure 1C). However, tiliroside treatment significantly reduced Caspase-1 p20 maturation and IL-1β cleavage compared with vehicle controls, indicating that tiliroside attenuates the activation of NLRP3 inflammasome (Figure 1C–E). Notably, the expression levels of NLRP3, pro-Caspase-1, and pro-IL-1β proteins were relatively unaffected by tiliroside treatment (Figure 1C and Figure S1). These results implicated that tiliroside-induced NLRP3 inflammasome inhibition is not mediated by regulating the protein expression of NLRP3 inflammasome components. Similar results were also obtained using mouse bone marrow-derived macrophages (BMDMs) pretreated with vehicle or tiliroside followed by LPS plus ATP stimulation (Supplementary Figure S2). Upon NLRP3 inflammasome activation, the adaptor ASC assembles into a large protein complex, which is termed “ASC speck” [22]. Thus, the ASC speck formation was examined to further evaluate the effect of tiliroside on NLRP3 inflammasome activation. Immunofluorescence staining of ASC showed the formation of specks in THP-1 macrophages primed with LPS and activated by ATP (Figure 1F,G). Nevertheless, tiliroside significantly decreased the frequency of cells with ASC specks, confirming its inhibitory role in activating the NLRP3 inflammasome (Figure 1F,G). Since inflammasome-mediated mature IL-1β plays a critical role in the induction of inflammatory factors and cytokines [9,11], the effect of tiliroside on the regulation of pro-inflammatory mediators in LPS plus ATP-stimulated THP-1 macrophages was assessed. Quantitative real-time PCR (qRT-PCR) analysis showed that the expression of *IL-1β*, *IL-6*, *TNFA*, and *IL-8* was notably elevated by LPS plus ATP, and these effects were primarily reversed by tiliroside treatment (Figure 1H). Importantly, gene expression of these pro-inflammatory cytokines and chemokines was also significantly diminished by a NLRP3 specific inhibitor MCC950. Co-treatment of cells with tiliroside and MCC950 showed no synergistic or additive effect, indicating that tiliroside dampens inflammatory responses by suppressing the NLRP3 inflammasome (Supplementary Figure S3). Taken together, these data suggest that tiliroside has the potential to attenuate NLRP3 inflammasome activation in macrophages.

### 2.2. Tiliroside Alleviates Mitochondrial Damage and Promotes AMPK Activation in Macrophages

**Figure 2:**
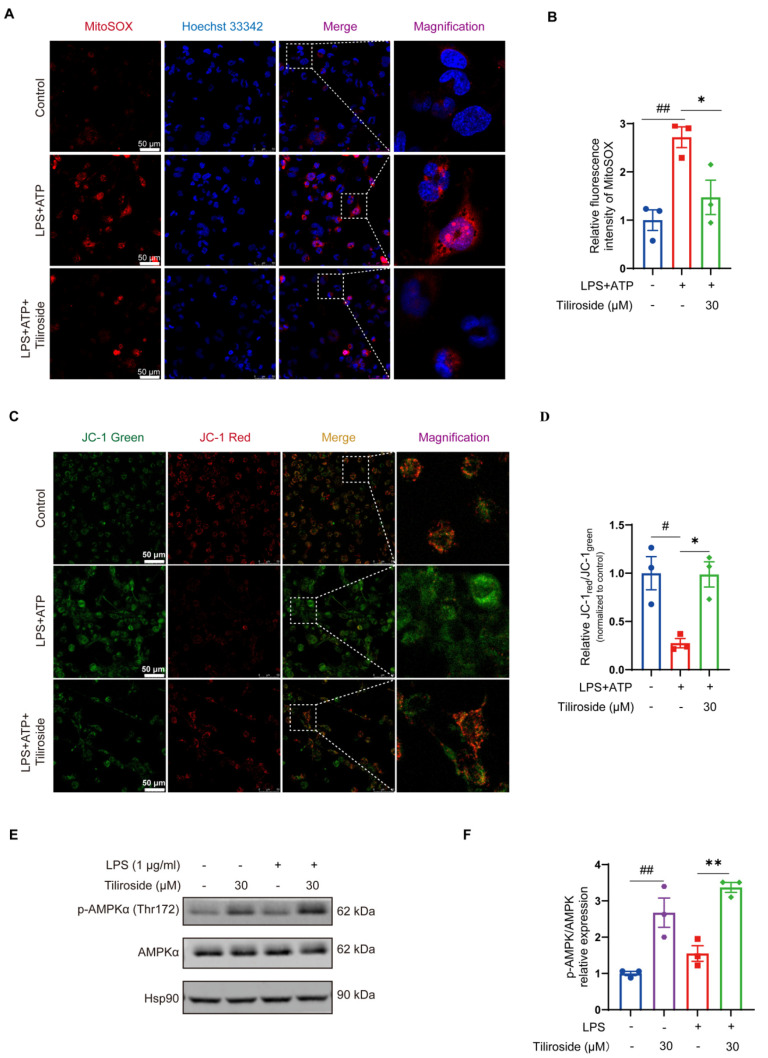
Tiliroside improves mitochondrial damage and regulates AMPK activation in macrophages. (**A**–**D**) THP-1 macrophages were pretreated with tiliroside (30 μM) or a vehicle for 24 h and then stimulated with LPS (2 μg/mL) for 5 h, followed by ATP (5 mM) treatment for 45 min. Representative images of THP-1 macrophages labeled with MitoSOX for mitochondrial ROS detection (red, MitoSOX; blue, Hoechst; scale bar, 50 μm) (**A**). Quantification of relative fluorescence intensity of MitoSOX (n = 3) (**B**). Representative images of THP-1 macrophages stained with JC-1 dye for mitochondrial membrane potential analysis (green, monomeric form of JC-1; red, multimeric form of JC-1; scale bar, 50 μm) (**C**). Mitochondrial membrane potential was quantified as the ratio of JC-1 red to JC-1 green (n = 3) (**D**). (**E**,**F**) THP-1 macrophages were pretreated with tiliroside (30 μM) or a vehicle for 24 h and then stimulated with or without LPS (1 μg/mL) for 1 h. Protein levels of p-AMPKα (Thr172) and AMPKα were determined by Western blotting (**E**) and quantification of p-AMPKα (Thr172) normalized to total AMPKα (n = 3) (**F**). Data are mean ± SEM. ^#^ *p* < 0.05, ^##^ *p* < 0.01, * *p* < 0.05, ** *p* < 0.01.

Next, the mechanism by which tiliroside blocks NLRP3 inflammasome activation was explored. As mentioned above, tiliroside did not modulate the expression of NLRP3 and pro-IL-1β in LPS plus ATP-stimulated macrophages (Figure 1C and Figure S1), indicating that tiliroside is not involved in regulating the priming step of NLRP3 inflammasome activation. Mitochondrial damage characterized by excessive reactive oxygen species (ROS) production and changes in mitochondrial membrane potential acts as a critical upstream signaling event of NLRP3 inflammasome activation [23,24,25]. Therefore, it was determined whether tiliroside affects mitochondrial ROS production during NLRP3 inflammasome activation by using MitoSOX (a mitochondrial superoxide indicator) as a specific fluorescent probe. Our data showed that LPS plus ATP induced a marked increase in MitoSOX-labeling mitochondrial ROS in THP-1 macrophages (Figure 2A,B). However, tiliroside treatment significantly mitigated LPS plus ATP-stimulated mitochondrial ROS production (Figure 2A,B). Since the accumulation of mitochondrial ROS is caused by mitochondrial dysfunction manifesting downregulated mitochondrial membrane potential [25], the effect of tiliroside on mitochondrial membrane potential in the presence of LPS plus ATP stimulation was then analyzed. JC-1 staining showed that LPS plus ATP resulted in a remarkable reduction of JC-1_red_:JC–1_green_ ratio, indicating that the mitochondrial membrane potential collapsed after stimulation (Figure 2C,D). Notably, this effect was reversed by tiliroside treatment, suggesting that tiliroside improves LPS plus ATP-induced decrease of mitochondrial membrane potential (Figure 2C,D). These results demonstrate that tiliroside plays a protective role in alleviating mitochondrial damage.

AMP-activated protein kinase (AMPK) has emerged as a central regulator of mitochondrial biology and homeostasis [26]. It was thus hypothesized that AMPK is involved in tiliroside’s beneficial role in preventing mitochondrial damage and NLRP3 inflammasome activation. Therefore, the effect of tiliroside on the activation of AMPK in THP-1 macrophages was examined. As determined by Western blot analysis, treatment of tiliroside prominently increased the phosphorylation of the AMPKα catalytic subunit at Thr-172 regardless of LPS stimulation (Figure 2E,F), indicating that tiliroside serves as an activator of AMPK in macrophages. It was predicted that AMPK may be a crucial mediator linking tiliroside and its pharmacological effects on mitochondrial damage and NLRP3 inflammasome activation in macrophages.

### 2.3. AMPK Is Required for Tiliroside-Mediated NLRP3 Inflammasome Inhibition and Mitochondrial Damage Amelioration

**Figure 3:**
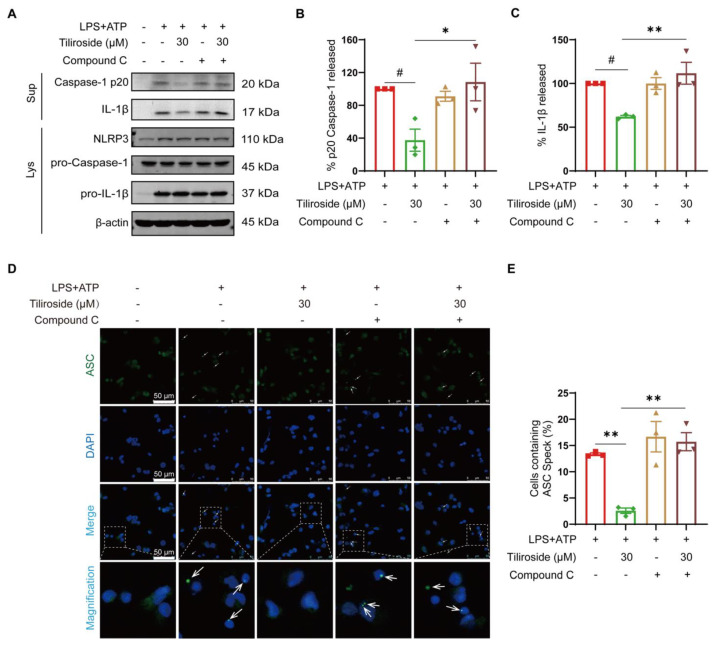
Tiliroside attenuates NLRP3 inflammasome activation in an AMPK-dependent manner. THP-1 macrophages were pretreated with tiliroside (30 μM) for 22 h, followed by co-treatment with Compound C for an additional 2 h, and then stimulated with LPS (2 μg/mL) for 5 h, followed by ATP (5 mM) treatment for 45 min. (**A**) Protein levels of Caspase-1 p20 and IL-1β in culture supernatants (SN) and NLRP3, pro-Caspase-1, and pro-IL-1β in cell lysates (Lys) of THP-1 macrophages were determined by Western blotting. (**B**,**C**) Relative Caspase-1 activation is shown as % of p20 release to the culture medium (n = 3) (**B**), and IL-1β maturation is shown as % of cleaved IL-1β release to the culture medium (n = 3) (**C**). (**D**) ASC specks were detected by immunofluorescence staining using anti-ASC (green) and counterstaining using DAPI (blue). The arrows indicate ASC specks (scale bar, 50 μm). (**E**) Quantification of the percentage of cells with ASC speck formation (n = 3). Data are mean ± SEM. ^#^ *p* < 0.05, * *p* < 0.05, ** *p* < 0.01.

To test our hypothesis mentioned above, the role of AMPK in tiliroside-mediated NLRP3 inflammasome inhibition and mitochondrial damage amelioration was further determined. An AMPK inhibitor Compound C was used to block the tiliroside-induced AMPK activation in THP-1 macrophages (Supplementary Figure S4), and the effect of tiliroside on LPS plus ATP-induced Caspase-1 p20 maturation and IL-1β cleavage with or without Compound C was analyzed. Our results showed that tiliroside decreased Caspase-1 p20 and mature IL-1β in cell supernatants, an effect that was abolished by Compound C (Figure 3A–C). In addition, Compound C did not affect NLRP3, pro-Caspase-1, and pro-IL-1β protein levels (Figure 3A and Figure S5). It was also asked whether AMPK contributed to the tiliroside-mediated regulation of ASC speck formation. Immunofluorescence analysis revealed that treatment of Compound C abrogated the tiliroside-mediated reduction of ASC specks formation (Figure 3D,E). Collectively, these findings indicate that tiliroside attenuates NLRP3 inflammasome activation in an AMPK-dependent manner.

**Figure 4:**
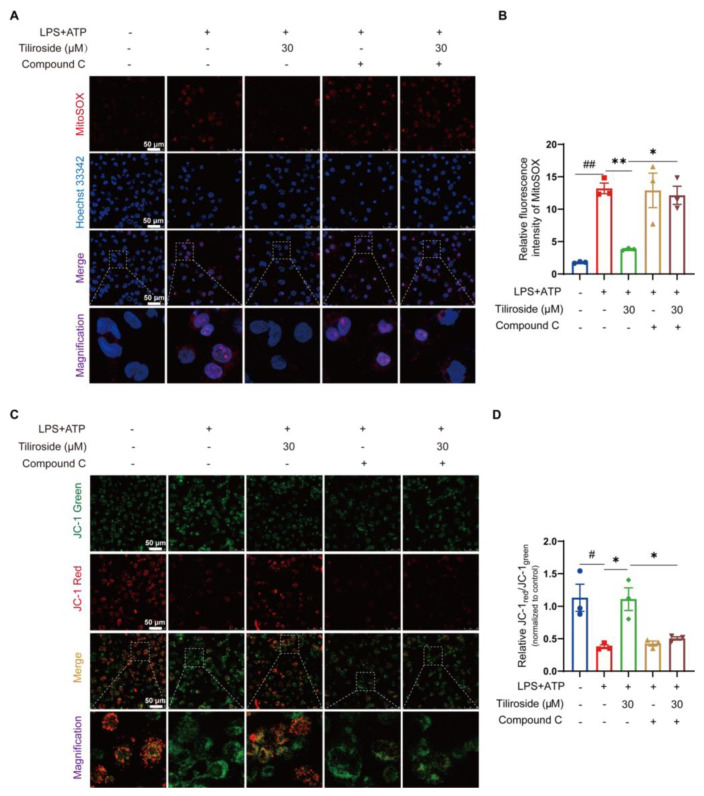
Tiliroside ameliorates mitochondrial damage during NLRP3 inflammasome activation depending on AMPK. THP-1 macrophages were pretreated with tiliroside (30 μM) for 22 h, followed by co-treatment with Compound C for an additional 2 h, and then stimulated with LPS (2 μg/mL) for 5 h, followed by ATP (5 mM) treatment for 45 min. (**A**) Representative images of THP-1 macrophages labeled with MitoSOX for mitochondrial ROS detection (red, MitoSOX; blue, Hoechst; scale bar, 50 μm). (**B**) Quantification of relative fluorescence intensity of MitoSOX (n = 3). (**C)** Representative images of THP-1 macrophages stained with JC-1 dye for mitochondrial membrane potential analysis (green, monomeric form of JC-1; red, multimeric form of JC-1; scale bar, 50 μm). (**D**) Mitochondrial membrane potential as the ratio of JC-1 red to JC-1 green was quantified (n = 3). Data are mean ± SEM. ^#^ *p* < 0.05, ^##^ *p* < 0.01, * *p* < 0.05, ** *p* < 0.01.

Next, the role of AMPK in ameliorating mitochondrial damage mediated by tiliroside in LPS plus ATP-stimulated macrophages was investigated. MitoSOX labeling analysis was performed to examine the possible involvement of AMPK in the tiliroside-mediated modulation of mitochondrial ROS production. As a result, it was observed that treatment with Compound C completely blocked the tiliroside-mediated attenuation of mitochondrial ROS production (Figure 4A,B). In addition, JC-1 staining for mitochondrial membrane potential detection also showed that the improvement effect of tiliroside on mitochondrial dysfunction could be reversed by Compound C (Figure 4C,D). These results demonstrate that tiliroside prevents mitochondrial damage during NLRP3 inflammasome activation depending on AMPK.

Based on the above findings, it is conceivable that tiliroside targets AMPK to protect against mitochondrial damage and suppress NLRP3 inflammasome activation in macrophages.

### 2.4. Tiliroside Protects Mice against Acute Lung Injury and Inhibits NLRP3 Inflammasome Activation In Vivo

**Figure 5:**
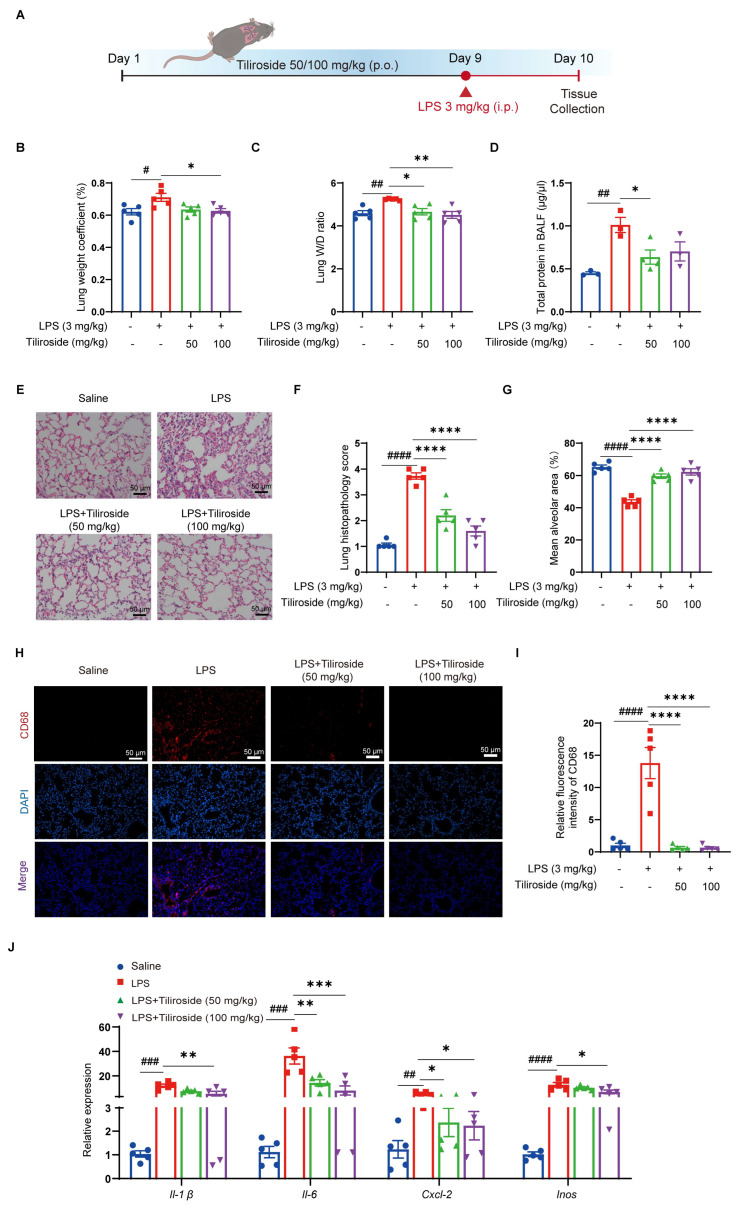
Tiliroside protects against LPS-induced acute lung injury in mice. (**A**) Schematic view of LPS-induced acute lung injury mouse model and the treatment regimen. (**B**,**C**) Lung-weight (g)-to-body-weight (g) ratio × 100% (n = 5) (**B**) and the lung wet/dry (W/D) weight ratio (n = 5) (**C**). (**D**) The protein concentration in BALF supernatants was measured (n = 3). (**E**) Representative H&E staining of mouse lung sections (scale bar, 50 μm). (**F**) Quantitative analysis of the lung histopathological changes based on H&E staining results (n = 5). (**G**) The mean alveolar area was quantified (n = 5). (**H**) Representative immunofluorescence staining of CD68 (red) in mouse lung tissues (scale bar, 50 μm). (**I**) Quantification of relative fluorescence intensity of CD68 (n = 5). (**J**) Gene expression levels of *Il-1β*, *Il-6*, *Cxcl-2*, and *Inos* in the lung tissues were analyzed by qRT-PCR (n = 5). Data are mean ± SEM. ^#^ *p* < 0.05, ^##^ *p* < 0.01, ^###^ *p* < 0.001, ^####^ *p* < 0.0001, * *p* < 0.05, ** *p* < 0.01, *** *p* < 0.001. **** *p* < 0.0001.

Given that tiliroside attenuates NLRP3 inflammasome activation *in vitro*, we then validated this finding under more physiologically relevant conditions using a mouse model of acute lung injury that has been reported to be NLRP3 inflammasome dependent [27]. Mice pretreated with tiliroside or vehicle were intraperitoneally injected with LPS to induce acute inflammatory lung injury (Figure 5A). Pulmonary edema, as indicated by the lung-to-body weight ratio, the wet-lung-to-dry-lung weight (W/D) ratio, and the total protein concentrations in bronchoalveolar lavage fluid (BALF), was significantly increased in vehicle-treated mice upon LPS challenge (Figure 5B–D). However, this response to LPS was blunted by tiliroside administration (Figure 5B–D). Moreover, histological analysis indicated that inflammatory lesions and tissue injuries (including disrupted pulmonary architecture, thickened alveolar septa, massive inflammatory cell infiltration, and interstitial and intra-alveolar edema) were obvious in lungs of vehicle-treated mice in response to LPS, and such pathological changes were alleviated by tiliroside administration (Figure 5E–G). The extent of inflammation was determined by macrophage infiltration and the expression of pro-inflammatory mediators in the lung tissues. Immunofluorescence staining of CD68 showed that a large number of macrophages were accumulated in LPS-induced vehicle control lungs, whereas such inflammatory response was repressed by tiliroside treatment (Figure 5H,I). Consistently, pro-inflammatory mediators *Il-1β*, *Il-6*, *Cxcl-2,* and *Inos* displayed high expression levels in LPS-challenged lungs of vehicle controls (Figure 5J). In contrast, tiliroside administration significantly attenuated the activation of these proinflammatory genes (Figure 5J). Collectively, tiliroside exerts protective effects on LPS-induced inflammatory lung injury in mice.

**Figure 6:**
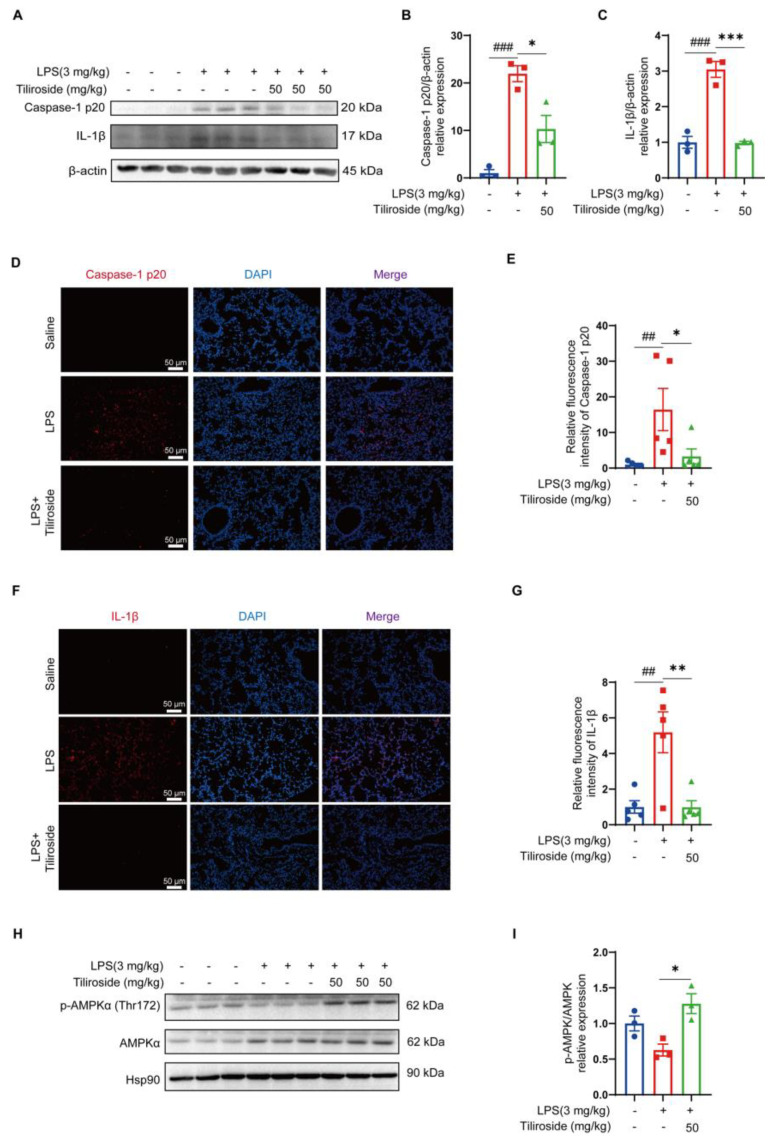
Tiliroside attenuates NLRP3 inflammasome activation *in vivo*. LPS-induced inflammatory lung injury and tiliroside administration (50 mg/kg) were performed as in Figure 5. (**A**–**C**) Western blotting analysis of Caspase-1 p20 and IL-1β in lung tissue samples (**A**), and quantification of matured Caspase-1 p20 (**B**) and IL-1β (**C**) protein levels (n = 3). (**D**–**G**) Representative immunofluorescence staining of Caspase-1 p20 (red) (**D**) and IL-1β (red) (**F**) in mouse lung tissues (scale bar, 50 μm), and quantification of relative fluorescence intensity of Caspase-1 p20 (**E**) and IL-1β (**G**) (n = 5). (**H**,**I**) Protein levels of p-AMPKα (Thr172) and AMPKα were determined by Western blotting (**H**) and quantification of p-AMPKα (Thr172) normalized to total AMPKα (n = 3) (**I**). Data are mean ± SEM. ^##^ *p* < 0.01, ^###^ *p* < 0.001, * *p* < 0.05, ** *p* < 0.01, *** *p* < 0.001.

Next, the pharmacological activities of tiliroside on NLRP3 inflammasome activation *in vivo* were assessed. Intraperitoneal injection of LPS elicited an NLRP3 inflammasome-dependent lung inflammation [27]. As expected, the LPS challenge promoted the maturation of pro-Caspase-1 to Caspase-1 p20 and the cleavage of pro-IL-1β to IL-1β in mouse lung tissues (Figure 6A–C). Notably, tiliroside significantly dampened the production of mature Caspase-1 p20 and IL-1β (Figure 6A–C). Consistently, immunofluorescence analysis also confirmed that tiliroside administration suppressed the production of Caspase-1 p20 and IL-1β in LPS-challenged lung tissues (Figure 6D–G). Thus, these results indicate that tiliroside attenuates NLRP3 inflammasome activation *in vivo*. Furthermore, the phosphorylation of AMPK in mouse lungs was markedly induced by the administration of tiliroside (Figure 6H,I), which is consistent with our *in vitro* observation showing tiliroside promotes AMPK activation (Figure 2E,F). Together with the critical role of AMPK in mediating tiliroside’s pharmacological actions *in vitro*, these data support an AMPK-dependent mechanism by which tiliroside preserves against mitochondrial stress, contributing to NLRP3 inflammasome inhibition and thereby benefiting NLRP3-related inflammatory disease, *in vivo*.

## 3. Discussion

**Figure 7:**
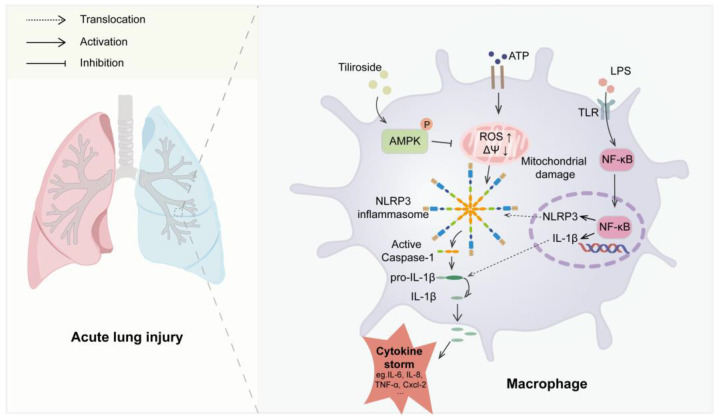
A proposed model for the role of tiliroside in regulating NLRP3 inflammasome activation and NLRP3-related disease. Tiliroside promotes AMPK activation in macrophages, thereby ameliorating mitochondrial damage by reducing mitochondrial ROS production and improving mitochondrial membrane potential (ΔΨ), dampening NLRP3 inflammasome activation and suppressing inflammatory lung injury in mice.

Tiliroside is a natural flavonoid with remarkable anti-inflammatory activities both *in vitro* and *in vivo* [17,18,19,20,21]. However, whether tiliroside regulates the activation of NLRP3 inflammasome remains elusive, which has been implicated in a wide array of human diseases. In this study, it was demonstrated for the first time that tiliroside attenuates NLRP3 inflammasome activation in macrophages, resulting in reduced inflammatory responses and protecting against LPS-induced acute lung injury in mice. Mechanistically, tiliroside was found to promote AMPK activation in macrophages, leading to ameliorated mitochondrial damage as evidenced by the improvement of mitochondrial ROS production and mitochondrial membrane potential, which is accompanied by the blockade of the activation of the NLRP3 inflammasome (Figure 7). Our findings unravel a novel pharmacological mechanism for tiliroside in repressing inflammation and shed light on the clinical application of tiliroside for managing NLRP3-related diseases.

NLRP3 inflammasome is a multiprotein complex responsible for the activation of Caspase-1 and subsequent maturation of IL-1β [3]. By using THP-1-derived macrophages primed with LPS followed by ATP stimulation, our data showed that pretreatment with tiliroside attenuated the maturation of pro-Caspase-1 to Caspase-1 p20 and the cleavage of pro-IL-1β to IL-1β (Figure 1C–E), suggesting an inhibitory role of tiliroside in NLRP3 inflammasome activation. Upon activation and assembly of NLRP3 inflammasome, ASC polymerizes in a prion-like fashion and forms a large, micrometer-sized, perinuclear structure termed the “ASC speck” that serves as a signal transduction platform for enhanced cytokine maturation by Caspase-1 [28]. Thus, the formation of ASC speck is recognized as a readout for NLRP3 inflammasome activation. Consistent with the notion that tiliroside inhibits the activation of NLRP3 inflammasome, it was observed that the LPS plus ATP-induced ASC speck formation was substantially mitigated by tiliroside treatment (Figure 1F,G). Therefore, these data further extend our understanding of tiliroside as an agent against inflammation. Additionally, the classical activated pro-inflammatory macrophages are characterized by NLRP3 inflammasome activation and the expression of various pro-inflammatory mediators [29,30,31]. In fact, NLRP3 inflammasome-induced IL-1β plays a causal role in amplifying the inflammatory response by stimulating cytokine production and recruiting additional immune mediators [9,11]. In our study, induction of the pro-inflammatory macrophage signature cytokines *IL-1β*, *IL-6*, *TNFA*, and *IL-8* were reduced by tiliroside in the THP-1 macrophage model of NLRP3 inflammasome activation (Figure 1H). These data are consistent with our previous finding that tiliroside represses M1 macrophage polarization [21] and indicate that NLRP3 inflammasome inhibition may be involved in tiliroside-mediated regulation of the M1 macrophage phenotype. Indeed, using NLRP3-specific inhibitor MCC950, it was found that the combination of MCC950 and tiliroside did not have an additive effect on diminishing expression levels of these M1 cytokines (Supplementary Figure S3). These results suggest that tiliroside attenuates NLRP3 inflammasome activation in macrophages to exert anti-inflammatory activity. Notably, mature IL-1β release was significantly reduced by tiliroside at this time point (Figure 1C,E). Furthermore, previous studies by others have shown that IL-1β production correlates with macrophage inflammatory responses, promoting the expression of inflammatory factors and cytokines, including IL-6, TNF-α, CXCL10, IL-11, IL-13, and CCL2 [11]. Thus, we speculate that the attenuation of pro-inflammatory gene expression by tiliroside in Supplementary Figure S3 is correlated with reduced IL-1β production. We thus conclude, and our results strongly suggest, that tiliroside inhibits NLRP3 inflammasome activation, thereby reducing mature IL-1β production, subsequently contributing to further attenuation of pro-inflammatory gene expression, a feed-forward loop. Given the known role of tiliroside in inhibiting the NF-κB inflammatory signaling pathway [18,19], our data indicate that tiliroside suppresses macrophage inflammatory response though both reducing NLRP3 inflammasome activation and the NF-κB inflammatory signaling pathway.

The general mechanisms underlying NLRP3 inflammasome activation involve both priming signaling (signal 1) and activation signaling (signal 2). The priming signaling is mediated by the activation of classic PRRs, such as the Toll-like receptor or tumor necrosis factor receptor, which then results in NF-κB-dependent upregulation of NLRP3 and pro-IL-1β [3,4]. The activation signaling is triggered by various PAMP or DAMP stimulation, including extracellular ATP, pore-forming toxins, RNA viruses, and crystal particulates. These stimuli further induce complicated cellular and molecular responses, including mitochondrial damage, ion flux, and lysosomal disruption, which subsequently promote the assembly and activation of the NLRP3 inflammasome [3,4]. In this study, it was shown that tiliroside did not affect NLRP3 and pro-IL-1β protein levels in LPS plus ATP-induced macrophages (Figure 1C and Figure S1), indicating that tiliroside has no noticeable role in regulating the NF-κB-dependent priming step (signal 1) during NLRP3 inflammasome activation. Notably, previous studies have demonstrated that tiliroside exerts an inhibitory effect on NF-κB activation upon inflammatory insult [18,19]. Thus, our results implicated that tiliroside regulates NLRP3 and pro-IL-1β independent of the NF-κB signaling pathway. Since tiliroside is not involved in modulating priming signaling-mediated NLRP3 and pro-IL-1β expression (signal 1), it was proposed that tiliroside may target the activation signaling (signal 2) to inactivate the NLRP3 inflammasome.

Emerging evidence indicates that mitochondria are central organelles controlling NLRP3 inflammasome activation [3]. In the context of mitochondrial stress, damaged mitochondria act as critical drivers for activating NLRP3 inflammasome through excessive production of mitochondrial ROS, the release of mitochondrial DNA into the cytosol, defective mitochondrial dynamics, etc. [3,23,24,25]. In the current study, it was observed that tiliroside mitigates mitochondrial ROS production and improves mitochondrial membrane potential in LPS plus ATP-induced macrophages (Figure 2A–D), indicating the protective effect of tiliroside on mitochondrial damage, which might be related to the mitigated NLRP3 inflammasome activation. Mitochondrial ROS has been demonstrated to be critical for activating the NLRP3 inflammasome. Abundant reports have illustrated that multiple danger or microbial signals such as small molecules targeting mitochondria, oxidized phosphatidylcholine, human respiratory syncytial virus, and supraphysiological testosterone can lead to increased mitochondrial ROS generation to promote NLRP3 inflammasome activation [32,33,34,35]. In contrast, treatment of mitochondrial ROS scavengers or inhibitors abolishes the activation of the NLRP3 inflammasome [29,36,37]. Furthermore, as an indicator of mitochondrial damage, loss of mitochondrial membrane potential is also linked to NLRP3 inflammasome activation partly through the apoptosis-related mechanism [24]. Therefore, it can be further speculated that the tiliroside-mediated decreased mitochondrial ROS and improved mitochondrial membrane potential may contribute to the attenuation of NLRP3 inflammasome activation. However, it is noteworthy that we cannot exclude that the protective effect of tiliroside on mitochondrial function may be due to the inhibition of NLRP3 inflammasome activation. Therefore, further studies are warranted to substantiate the relationship between mitochondrial protection and tiliroside-mediated NLRP3 inflammasome inhibition.

AMPK is an evolutionarily conserved serine/threonine kinase that plays fundamental roles in regulating cellular adaptation to energetic stress. More importantly, accumulating evidence suggests that AMPK is a central regulator of mitochondrial function, maintaining mitochondrial homeostasis in response to stress signals [26]. It is postulated that tiliroside may inhibit NLRP3 inflammasome activation through an AMPK-dependent manner. Our results showed that AMPK phosphorylation was markedly increased in tiliroside-treated macrophages (Figure 2E,F), and pharmacological inhibition of AMPK abrogated tiliroside-mediated NLRP3 inflammasome attenuation (Figure 3). Concurrently, the beneficial effects of tiliroside on mitochondrial ROS production and mitochondrial membrane potential were also reversed by AMPK inhibition (Figure 4). These findings provide evidence demonstrating that tiliroside promotes AMPK activation, thereby ameliorating mitochondrial ROS production, improving mitochondrial membrane potential, and inhibiting NLRP3 inflammasome activation. It is noteworthy that the AMPK inhibitor Compound C did not affect NLRP3 and pro-IL-1β protein levels (Figure 3A and Figure S5), suggesting that in THP-1 cells, inhibition of AMPK has little effect on the NF-κB-dependent priming signaling (signal 1), which is in accordance with previously published data [38,39,40]. Intriguingly, a recent study has revealed a dynamic relationship between AMPK and mitochondrial ROS. On the one hand, mitochondrial ROS serves as a physiological activator of AMPK [41]. On the other hand, activated AMPK in turn triggers a PGC-1α-dependent antioxidant response that limits mitochondrial ROS production [41]. It is thus reasonable to speculate that tiliroside may physiologically upregulate the mitochondrial ROS level, which subsequently promotes AMPK activation and thereby induces the intracellular antioxidant pathway to suppress LPS plus ATP-induced overproduction of mitochondrial ROS, ultimately leading to NLRP3 inflammasome inhibition. Nevertheless, this hypothesis needs further validation. In addition to the AMPK/mitochondrial damage pathway, whether additional mechanisms such as ion flux, lysosomal disruption, and posttranslational modifications of inflammasome components also contribute to tiliroside-mediated NLRP3 inflammasome inhibition remains unknown, and thus are warranted future investigations. Furthermore, in addition to NLRP3 inflammasome, there are several other types of inflammasomes, including NLRP1, NLRC4, and AIM2, that have been reported [42]. Whether tiliroside regulates these inflammasomes has yet to be determined.

Aberrant NLRP3 inflammasome activation is involved in the pathogenesis of various diseases [7]. Since tiliroside attenuates NLRP3 inflammasome activation *in vitro*, its pharmacological activities *in vivo* were examined and its therapeutic potential in NLRP3-related disease was evaluated. In this regard, a mouse model of LPS-induced acute lung injury that is NLRP3 inflammasome dependent was used [43,44,45]. In the present study, it was shown that oral administration of tiliroside significantly decreased pulmonary edema, alleviated lung tissue pathological changes, and repressed inflammatory cell infiltration and gene expression of pro-inflammatory mediators in LPS-challenged mouse lungs (Figure 5), implying the protective effects of tiliroside on LPS-induced inflammatory lung injury. Importantly, our *in vivo* data indicates that tiliroside attenuates NLRP3 inflammasome activation through the AMPK signaling pathway (Figure 6), consistent with the *in vitro* findings. Since NLRP3 inflammasome has been reported to play a causal role in LPS-induced acute lung injury [43,44,45], it was speculated that the beneficial effects of tiliroside *in vivo* rely on its suppressive activity for the NLRP3 inflammasome, leading to the attenuation of inflammatory responses and the inhibition of lung injury in mice. In recent years, many small molecules with potential inhibitory effects on NLRP3 inflammasome activation have been reported, and some of them have demonstrated promising therapeutic potential [7]. However, none of them have been currently approved for use in the clinic [7]. Evidence has shown that dietary polyphenols including flavonoids render various beneficial effects on human health without raising safety concerns [16]. Remarkably, tiliroside is a flavonoid contained in several edible plants or specific plant parts (fruits, leaves, or roots), and these plant materials rich in tiliroside are widely used as both food and medicines and are employed in human health care [16]. Together with our current study showing tiliroside’s protective role in attenuating NLRP3 inflammasome activation both *in vivo* and *in vitro*, it is tempting to expect that tiliroside may represent a potential therapeutic agent for managing NLRP3-mediated inflammatory diseases.

## 4. Materials and Methods

### 4.1. Chemicals and Reagents

Tiliroside (≥98% pure) was obtained from Chengdu Derick Biotechnology Co., Ltd. (drk-0667, Chengdu, China). Dimethyl sulfoxide (DMSO) was purchased from Solarbio (D8370, Beijing, China). Sodium carboxymethyl cellulose (CMC-Na) was obtained from Xilong Scientific (10106701, Guangzhou, China). LPS (*Escherichia coli*, O111:B4) was purchased from Sigma-Aldrich (L2630, St. Louis, MO, USA). ATP was purchased from Roche Diagnosis Ltd. (10519979001, Basel, Switzerland). Phorbol-12-myristate-13-acetate (PMA, HY-18739) and Compound C (HY-13418) were purchased from MedChemExpress (Middlesex, NJ, USA). MCC950 was purchased from Selleck (S7809, Houston, TX, USA). MitoSOX™ Red Mitochondrial Superoxide Indicators (M36008) and TRIzol reagent (15596-018) were purchased from Invitrogen (Carlsbad, CA, USA). The JC-1 Assay Kit (C2003S) and Hoechst 33342 (C1028) were purchased from Beyotime Biotechnology (Nanjing, China). RPMI-1640 cell culture medium (11875500BT), the Pierce™ BCA Protein Assay Kit (23225), and Pierce™ ECL reagent (32106) were purchased from Thermo Fisher Scientific (Waltham, MA, USA). The CCK-8 Assay Kit (40203ES60), Hifair™ III 1st Strand cDNA Synthesis SuperMix (11141ES60), and Hieff^®^ qPCR SYBR Green Master Mix (11201ES08) were purchased from YEASEN Biotech Co. Ltd. (Shanghai, China). Primary antibodies against NLRP3 (AG-20B-0014) and Caspase-1 (AG-20B-0042) were obtained from AdipoGen Life Sciences (San Diego, CA, USA). The antibody against cleaved IL-1β was purchased from Affinity Biosciences (AF4006, Changzhou, China). The antibody against pro-IL-1β was purchased from R&D systems (AF-401-NA, Minneapolis, MN, USA). The antibody against ASC was provided by Biolegend (653903, San Diego, CA, USA). Antibodies against Caspase-1 (#2225), AMPKα (#5831), and p-AMPKα (Thr172, #2535) were purchased from Cell Signaling Technology (Danvers, MA, USA). The antibody against CD68 was purchased from Bio-Rad (MCA1957, Hercules, CA, USA). HRP goat anti-rabbit IgG (H+L) (31460), HRP goat anti-mouse IgG (H+L) (31430), Alexa Fluor 594 goat anti-rat IgG (H+L) (A11008), and Alexa Fluor 594 goat anti-rabbit IgG (H+L) (R37117) were obtained from Invitrogen (Carlsbad, CA, USA). IRDye^®^ 680RD donkey anti-mouse IgG (926-68072) and IRDye^®^ 800CW donkey anti-rabbit IgG (926-32213) were provided by LI-COR (Lincoln, NE, USA).

### 4.2. Cell Culture and Stimulation

Human monocyte leukemia cell line (THP-1) was obtained from the China General Microbiological Culture Collection Center (Beijing, China). The cells were cultured at a density of 5 × 10^5^ cells/mL in RPMI 1640 medium supplemented with 10% fetal calf serum and 1% penicillin/streptomycin solution at 37 °C in a 5% CO_2_ incubator. In all experiments, THP-1 monocytes were treated with PMA (200 nM) to induce differentiation into adherent macrophages for 24 h before pharmacological intervention. For NLRP3 inflammasome activation, THP-1-derived macrophages were pretreated with tiliroside (3, 10, 30 μM) or a vehicle for 24 h, and then stimulated with LPS (2 μg/mL) for 5 h followed by ATP (5 mM) treatment for 45 min. Supernatants and cells were collected for subsequent analysis.

### 4.3. Cell Viability Assay

THP-1 macrophages were cultured in 96-well plates to reach sub-confluence, and then they were treated with different concentrations of tiliroside for 30 h. Subsequently, 10 μL of CCK-8 was added into each well to incubate cells for another 1 h at 37 °C with 5% CO_2_. Absorbances at 450 nm were measured using a microplate reader (Bio-Rad).

### 4.4. Western Blotting

Total proteins were isolated from mouse lung tissues or cultured macrophages, and the protein concentrations were determined using the Pierce™ BCA Protein Assay Kit. SDS-PAGE was performed to separate the protein extracts, and then they were transferred to PVDF membranes with a thickness of 0.45 μm (Millipore, Billerica, MA, USA). After being blocked for 1 h at room temperature with 5% BSA, the membranes were treated with primary antibodies overnight at 4 °C. Next, the membranes were incubated with corresponding secondary antibodies for 1 h followed by signal detection using Pierce™ ECL reagent or ODYSSEY Infrared Imaging System (LI-COR). Band intensities were quantified with ImageStudio software (LI-COR, Version 5.2). Hsp90 (Figure 1C), β-actin (Figure 3A, Figure 6A and Figure S2), or total AMPK (Figure 2E, Figure 6H and Figure S4), was used as a reference protein. All experiments were repeated three times.

### 4.5. RNA Extraction and qRT-PCR Assay

Total RNA was extracted from tissue homogenates or cultured cells using TRIzol reagent according to the manufacturer’s instructions. The RNA samples were then reverse transcribed into cDNA using Hifair™ III 1st Strand cDNA Synthesis SuperMix, and the resulting cDNA was amplified by qRT-PCR using Hieff^®^ qPCR SYBR Green Master Mix and the Roche LC480 Real-Time PCR System. β-actin was used as a loading control and the relative gene expression was determined using the comparative ΔΔCt method. The primer sequences for the qRT-PCR assay are listed in Supplementary Materials in Table S1.

### 4.6. Immunofluorescence

For tissue samples, paraffin slices were dewaxed and hydrated, processed for antigen restoration using EDTA buffer, and blocked with 10% goat serum at room temperature. For cultured cell samples, cells were fixed with 4% paraformaldehyde, permeabilized with 0.2% Triton X-100, and blocked with 5% goat serum. All samples were then treated with primary antibodies overnight at 4 °C. After washing in PBS, slides were incubated with corresponding secondary antibodies for 1 h and counter stained with DAPI. Images were captured using a Leica SP8 confocal microscope (Tokyo, Japan). Data were quantified with ImageJ software (Version 1.49).

### 4.7. Determination of Mitochondrial ROS Production

To determine mitochondrial ROS production, MitoSOX Red labeling was performed according to the manufacturer’s instructions. Briefly, THP-1 macrophages were incubated with MitoSOX Red (5 μM) for 10 min at 37 °C. After washing with HBSS, cells were counter-stained with Hoechst 33342, and the MitoSOX Red fluorescence was measured using a Leica SP8 confocal microscope. Data were quantified with ImageJ software (Version 1.49).

### 4.8. Measurement of Mitochondrial Membrane Potential

Mitochondrial membrane potential was measured using the JC-1 Assay Kit following the manufacturer’s protocol. Briefly, THP-1 macrophages were treated with freshly prepared JC-1 dye solution for 20 min at 37 °C and washed with JC-1 assay buffer. The fluorescence of multimeric form (JC-1 red) and monomeric form (JC-1 green) was measured using a Leica SP8 confocal microscope. Data were quantified with ImageJ software (Version 1.49).

### 4.9. Animals

C57BL/6 mice (male, 6–8 weeks old, 20 ± 3 g) were obtained from Hunan Slack Jingda Laboratory Animal Co., Ltd. (Changsha, China). The animal license number was SCXK (Xiang) 2019-0004 and the quality certificate number was 43072722012989723. All mice were housed under controlled conditions with an appropriate temperature (22–26 °C), humidity (50–60%), and 12 h/12 h light/dark cycle in a specific-pathogen-free environment and were provided with free access to food and water. All animal experimental procedures were approved by the Institutional Animal Care and Use Committee of the Jiangxi University of Chinese Medicine (TEMPOR20230035) and complied with all relevant ethical regulations.

### 4.10. Murine Model of LPS-Induced Acute Lung Injury

All mice were randomly divided into four groups (n = 5 per group): saline group, LPS group, LPS + tiliroside group (administered 50 mg/kg tiliroside), and LPS + tiliroside group (administered 100 mg/kg tiliroside). The tiliroside was dissolved in 0.5% CMC-Na and orally administered by gastric gavage for 9 days before induction of lung injury. An equal amount of 0.5% CMC-Na was administered simultaneously to the saline and LPS groups. The mouse model of LPS-induced acute lung injury was established by injecting intraperitoneally with a single dose of LPS (3 mg/kg). After 24 h, the mice were sacrificed, and tissues were harvested for subsequent experiment.

### 4.11. BALF Collection and Its Protein Concentration Analysis

Following the mouse sacrifice, the cervical trachea was completely exposed, 1 mL of pre-cooled phosphate buffer was injected into the alveoli through the trachea with a syringe, and BALF was collected after lingering for 1–2 min. The final BALF was harvested after the lung was rinsed three times with the recovered lavage fluid. BALF was centrifuged (300× *g*) for 10 min at 4 °C, and the supernatant of BALF was collected. The protein concentration in BALF supernatant was detected using the Pierce™ BCA Protein Assay Kit.

### 4.12. Lung Wet/Dry Weight Measurement

To determine the edema of the lung tissue, the upper left lung was removed, and the wet weight was immediately measured after the surface blood was removed from the tissue. The same lung tissue was placed in an oven at 60 °C for 48 h, and then weighted again to obtain the dry weight. Next, the lung wet-to-dry weight ratio was calculated.

### 4.13. Histopathological Analysis

Mouse lung tissues were fixed with 4% neutral buffered formalin for 24 h at 4 °C and then processed for paraffin embedding and sectioned at 5 μm in thickness. Lung sections were subjected to deparaffinization and hydration using a graded ethanol series, and hematoxylin and eosin (H&E) staining [46] was performed to analyze histopathological damage of the lung tissues. A semi-quantitative scoring criteria was used to evaluate lung injury based on interstitial inflammation, congestion, and edema, as previously described [47].

### 4.14. Statistical Analysis

Data are presented as mean ± standard errors of the means (SEM). Prism software (GraphPad, CA, USA, Version 8.0) was used for statistical analyses. Statistical differences were assessed by two-tailed Student’s *t*-test (two groups’ comparisons) and one-way ANOVA followed by the Tamhane T2 test (multiple groups’ comparisons). A value of *p* < 0.05 is considered statistically significant.

## 5. Conclusions

This study illustrated, for the first time, that tiliroside targets the AMPK pathway, ameliorating mitochondrial damage, attenuating NLRP3 inflammasome activation, and improving LPS-induced acute lung injury in mice. This study extends our understanding of tiliroside as a natural compound with anti-inflammatory activity and sheds light on the clinical use of tiliroside in preventing and treating NLRP3-related disease.

## Data Availability

The original contributions presented in this study are all included in the article and/or Supplementary Materials. Further inquiries can be directed to the corresponding authors.

## Supplementary Materials

The following supporting information can be downloaded at: https://www.mdpi.com/article/10.3390/molecules28227527/s1, Table S1: Sequences of qRT-PCR primers used in this study; Figure S1: The effect of tiliroside on the expression of NLRP3, pro-Caspase-1 and pro-IL-1β in LPS plus ATP-induced THP-1 macrophages; Figure S2: Tiliroside inhibits NLRP3 inflammasome activation in BMDMs; Figure S3: The effect of tiliroside on the expression of pro-inflammatory mediators can be blocked by NLRP3 inhibitor MCC950; Figure S4: Compound C blocks AMPK activation in THP-1 macrophages; Figure S5: The effect of Compound C on tiliroside-mediated regulation of NLRP3, pro-Caspase-1 and pro-IL-1β in LPS plus ATP-induced THP-1 macrophages.

## References

[1] Martinon F., Mayor A., Tschopp J. (2009). The inflammasomes: Guardians of the body. Annu. Rev. Immunol..

[2] Kelley N., Jeltema D., Duan Y., He Y. (2019). The NLRP3 Inflammasome: An Overview of Mechanisms of Activation and Regulation. Int. J. Mol. Sci..

[3] Paik S., Kim J.K., Silwal P., Sasakawa C., Jo E.-K. (2021). An update on the regulatory mechanisms of NLRP3 inflammasome activation. Cell. Mol. Immunol..

[4] Swanson K.V., Deng M., Ting J.P.-Y. (2019). The NLRP3 inflammasome: Molecular activation and regulation to therapeutics. Nat. Rev. Immunol..

[5] Davis B.K., Wen H., Ting J.P.-Y. (2011). The Inflammasome NLRs in Immunity, Inflammation, and Associated Diseases. Annu. Rev. Immunol..

[6] Jo E.-K., Kim J.K., Shin D.-M., Sasakawa C. (2016). Molecular mechanisms regulating NLRP3 inflammasome activation. Cell. Mol. Immunol..

[7] Zahid A., Li B., Kombe A.J.K., Jin T., Tao J. (2019). Pharmacological Inhibitors of the NLRP3 Inflammasome. Front. Immunol..

[8] Weber A., Wasiliew P., Kracht M. (2010). Interleukin-1 (IL-1) Pathway. Sci. Signal..

[9] Weber A., Wasiliew P., Kracht M. (2010). Interleukin-1beta (IL-1beta) processing pathway. Sci. Signal..

[10] Chousterman B.G., Swirski F.K., Weber G.F. (2017). Cytokine storm and sepsis disease pathogenesis. Semin. Immunopathol..

[11] Pan P., Shen M., Yu Z., Ge W., Chen K., Tian M., Xiao F., Wang Z., Wang J., Jia Y. (2021). SARS-CoV-2 N protein promotes NLRP3 inflammasome activation to induce hyperinflammation. Nat. Commun..

[12] Sanchez-Lopez E., Zhong Z., Stubelius A., Sweeney S.R., Booshehri L.M., Antonucci L., Liu-Bryan R., Lodi A., Terkeltaub R., Lacal J.C. (2019). Choline Uptake and Metabolism Modulate Macrophage IL-1β and IL-18 Production. Cell Metab..

[13] Danielski L.G., Della Giustina A., Bonfante S., Barichello T., Petronilho F. (2020). The NLRP3 Inflammasome and Its Role in Sepsis Development. Inflammation.

[14] Bagherniya M., Khedmatgozar H., Fakheran O., Xu S., Johnston T.P., Sahebkar A. (2021). Medicinal plants and bioactive natural products as inhibitors of NLRP3 inflammasome. Phytother. Res..

[15] Yang J., Zhong C., Yu J. (2023). Natural Monoterpenes as Potential Therapeutic Agents against Atherosclerosis. Int. J. Mol. Sci..

[16] Grochowski D.M., Locatelli M., Granica S., Cacciagrano F., Tomczyk M. (2018). A Review on the Dietary Flavonoid Tiliroside. Compr. Rev. Food Sci. Food Saf..

[17] Jin X., Song S., Wang J., Zhang Q., Qiu F., Zhao F. (2016). Tiliroside, the major component of Agrimonia pilosa Ledeb ethanol extract, inhibits MAPK/JNK/p38-mediated inflammation in lipopolysaccharide-activated RAW 264.7 macrophages. Exp. Ther. Med..

[18] Velagapudi R., Aderogba M., Olajide O.A. (2014). Tiliroside, a dietary glycosidic flavonoid, inhibits TRAF-6/NF-κB/p38-mediated neuroinflammation in activated BV2 microglia. Biochim. Biophys. Acta.

[19] Velagapudi R., El-Bakoush A., Olajide O.A. (2018). Activation of Nrf2 Pathway Contributes to Neuroprotection by the Dietary Flavonoid Tiliroside. Mol. Neurobiol..

[20] Sala A., Recio M., Schinella G.R., Máñez S., Giner R.M., Cerdá-Nicolás M., Ríos J.-L. (2003). Assessment of the anti-inflammatory activity and free radical scavenger activity of tiliroside. Eur. J. Pharmacol..

[21] Zhuang H., Lv Q., Zhong C., Cui Y., He L., Zhang C., Yu J. (2021). Tiliroside Ameliorates Ulcerative Colitis by Restoring the M1/M2 Macrophage Balance via the HIF-1α/glycolysis Pathway. Front. Immunol..

[22] Nagar A., Rahman T., Harton J.A. (2021). The ASC Speck and NLRP3 Inflammasome Function Are Spatially and Temporally Distinct. Front. Immunol..

[23] Zhou R., Yazdi A.S., Menu P., Tschopp J. (2011). A role for mitochondria in NLRP3 inflammasome activation. Nature.

[24] Liu Q., Zhang D., Hu D., Zhou X., Zhou Y. (2018). The role of mitochondria in NLRP3 inflammasome activation. Mol. Immunol..

[25] Mishra S.R., Mahapatra K.K., Behera B.P., Patra S., Bhol C.S., Panigrahi D.P., Praharaj P.P., Singh A., Patil S., Dhiman R. (2021). Mitochondrial dysfunction as a driver of NLRP3 inflammasome activation and its modulation through mitophagy for potential therapeutics. Int. J. Biochem. Cell Biol..

[26] Wu S., Zou M.H. (2020). AMPK, Mitochondrial Function, and Cardiovascular Disease. Int. J. Mol. Sci..

[27] Feng Y., Li M., Yangzhong X., Zhang X., Zu A., Hou Y., Li L., Sun S. (2022). Pyroptosis in inflammation-related respiratory disease. J. Physiol. Biochem..

[28] Hoss F., Rolfes V., Davanso M.R., Braga T.T., Franklin B.S. (2018). Detection of ASC Speck Formation by Flow Cytometry and Chemical Cross-linking. Methods Mol. Biol..

[29] Wu K., Yuan Y., Yu H., Dai X., Wang S., Sun Z., Wang F., Fei H., Lin Q., Jiang H. (2020). The gut microbial metabolite trimethylamine N-oxide aggravates GVHD by inducing M1 macrophage polarization in mice. Blood.

[30] Tian J., Chang S., Wang J., Chen J., Xu H., Huang T., Wang J., Kang J., Fan W., Wang Y. (2023). S1P/S1PR1 axis promotes macrophage M1 polarization through NLRP3 inflammasome activation in Lupus nephritis. Mol. Immunol..

[31] Zhang J., Liu X., Wan C., Liu Y., Wang Y., Meng C., Zhang Y., Jiang C. (2020). NLRP3 inflammasome mediates M1 macrophage polarization and IL-1β production in inflammatory root resorption. J. Clin. Periodontol..

[32] Groß C.J., Mishra R., Schneider K.S., Médard G., Wettmarshausen J., Dittlein D.C., Shi H., Gorka O., Koenig P.A., Fromm S. (2016). K(+) Efflux-Independent NLRP3 Inflammasome Activation by Small Molecules Targeting Mitochondria. Immunity.

[33] Yeon S.H., Yang G., Lee H.E., Lee J.Y. (2017). Oxidized phosphatidylcholine induces the activation of NLRP3 inflammasome in macrophages. J. Leukoc. Biol..

[34] Bedient L., Pokharel S.M., Chiok K.R., Mohanty I., Beach S.S., Miura T.A., Bose S. (2020). Lytic Cell Death Mechanisms in Human Respiratory Syncytial Virus-Infected Macrophages: Roles of Pyroptosis and Necroptosis. Viruses.

[35] Alves J.V., da Costa R.M., Pereira C.A., Fedoce A.G., Silva C.A.A., Carneiro F.S., Lobato N.S., Tostes R.C. (2020). Supraphysiological Levels of Testosterone Induce Vascular Dysfunction via Activation of the NLRP3 Inflammasome. Front. Immunol..

[36] Dai J., Zhang X., Wang Y., Chen H., Chai Y. (2017). ROS-activated NLRP3 inflammasome initiates inflammation in delayed wound healing in diabetic rats. Int. J. Clin. Exp. Pathol..

[37] Yue H., Yang Z., Ou Y., Liang S., Deng W., Chen H., Zhang C., Hua L., Hu W., Sun P. (2021). Tanshinones inhibit NLRP3 inflammasome activation by alleviating mitochondrial damage to protect against septic and gouty inflammation. Int. Immunopharmacol..

[38] Deng Z., Ni J., Wu X., Wei H., Peng J. (2020). GPA peptide inhibits NLRP3 inflammasome activation to ameliorate colitis through AMPK pathway. Aging.

[39] Zhang H., Gong X., Ni S., Wang Y., Zhu L., Ji N. (2019). C1q/TNF-related protein-9 attenuates atherosclerosis through AMPK-NLRP3 inflammasome singling pathway. Int. Immunopharmacol..

[40] Tang G., Duan F., Li W., Wang Y., Zeng C., Hu J., Li H., Zhang X., Chen Y., Tan H. (2019). Metformin inhibited Nod-like receptor protein 3 inflammasomes activation and suppressed diabetes-accelerated atherosclerosis in apoE(−/−) mice. Biomed. Pharmacother. Biomed. Pharmacother..

[41] Rabinovitch R.C., Samborska B., Faubert B., Ma E.H., Gravel S.-P., Andrzejewski S., Raissi T.C., Pause A., St.-Pierre J., Jones R.G. (2017). AMPK Maintains Cellular Metabolic Homeostasis through Regulation of Mitochondrial Reactive Oxygen Species. Cell Rep..

[42] Broz P., Dixit V.M. (2016). Inflammasomes: Mechanism of assembly, regulation and signalling. Nat. Rev. Immunol..

[43] Jiang R., Xu J., Zhang Y., Zhu X., Liu J., Tan Y. (2021). Ligustrazine Alleviate Acute Lung Injury Through Suppressing Pyroptosis and Apoptosis of Alveolar Macrophages. Front. Pharmacol..

[44] Liu B., Wang Z., He R., Xiong R., Li G., Zhang L., Fu T., Li C., Li N., Geng Q. (2022). Buformin alleviates sepsis-induced acute lung injury via inhibiting NLRP3-mediated pyroptosis through an AMPK-dependent pathway. Clin. Sci..

[45] Ying Y., Mao Y., Yao M. (2019). NLRP3 Inflammasome Activation by MicroRNA-495 Promoter Methylation May Contribute to the Progression of Acute Lung Injury. Mol. Ther. Nucleic Acids.

[46] Guo Y., Liu Y., Zhao S., Xu W., Li Y., Zhao P., Wang D., Cheng H., Ke Y., Zhang X. (2021). Oxidative stress-induced FABP5 S-glutathionylation protects against acute lung injury by suppressing inflammation in macrophages. Nat. Commun..

[47] Li J., Lu K., Sun F., Tan S., Zhang X., Sheng W., Hao W., Liu M., Lv W., Han W. (2021). Panaxydol attenuates ferroptosis against LPS-induced acute lung injury in mice by Keap1-Nrf2/HO-1 pathway. J. Transl. Med..

