# Supplementary material for: Tiliroside Attenuates NLRP3 Inflammasome Activation in Macrophages and Protects against Acute Lung Injury in Mice
Source: Molecules. 2023 Nov 10;28(22):7527. doi: 10.3390/molecules28227527 (PMC10673355; doi:10.3390/molecules28227527)
Supplement: Supplementary file 1 [file molecules-28-07527-s001.zip › molecules-2629209-supplementary.pdf]

# Tiliroside Attenuates NLRP3 Inflammasome Activation in Macrophages and Protects against Acute Lung Injury in Mice

**Chao Zhong [1,†,*], Jing Yang [1,†], Keke Deng [1], Xiaoya Lang [1], Jiangtao Zhang [1], Min Li [2], Liang Qiu [1], Guoyue Zhong [2] and Jun Yu [3,*]**

[1] Center for Translational Medicine, College of Traditional Chinese Medicine, Jiangxi University of Chinese Medicine, Nanchang 330004, China
[2] Center for Traditional Chinese Medicine Resources and Ethnic Medicine, Jiangxi University of Chinese Medicine, Nanchang 330004, China
[3] Department of Cardiovascular Sciences and Center for Metabolic Disease Research, Lewis Katz School of Medicine, Temple University, Philadelphia, PA 19140, USA
* Correspondence: 20181012@jxutcm.edu.cn (C.Z.); jun.yu@temple.edu (J.Y.)
† These authors contributed equally to this work.

**Supplementary Table S1:** Sequences of qRT-PCR primers used in this study

| Gene | Species | Forward primer (5'-3') | Reverse primer (5'-3') |
|---|---|---|---|
| *ACTB* | *Homo* | CTCACCATGGATGATGATATCGC | AGGAATCCTTCTGACCCATGC |
| *IL6* | *Homo* | TGCAATAACCACCCCTGACC | GTGCCCATGCTACATTTGCC |
| *IL8* | *Homo* | AGCCTTCCTGATTTCTGCAG | GTCCACTCTCAATCACTCTCAG |
| *TNFA* | *Homo* | ACTTTGGAGTGATCGGCC | GCTTGAGGGTTTGCTACAAC |
| *CCL2* | *Homo* | TGTCCCAAAGAAGCTGTGATC | ATTCTTGGGTTGTGGAGTGAG |
| *Actb* | *Mus* | CTTTGCCACGGACGAGAC | TCATTGTACTCTGAGGGCTGAC |
| *Il-6* | *Mus* | GAACAACGATGATGCACTTGC | CTTCATGTACTCCAGGTAGCTATGGT |
| *Cxcl-2* | *Mus* | CCAACCACCAGGCTACAGG | GCGTCACACTCAAGCTCTG |
| *Il-1β* | *Mus* | AGTTGACGGACCCCAAAAG | AGCTGGATGCTCTCATCAGG |
| *Inos* | *Mus* | CTTTGCCACGGACGAGAC | TCATTGTACTCTGAGGGCTGAC |

**Supplementary Figure S1**

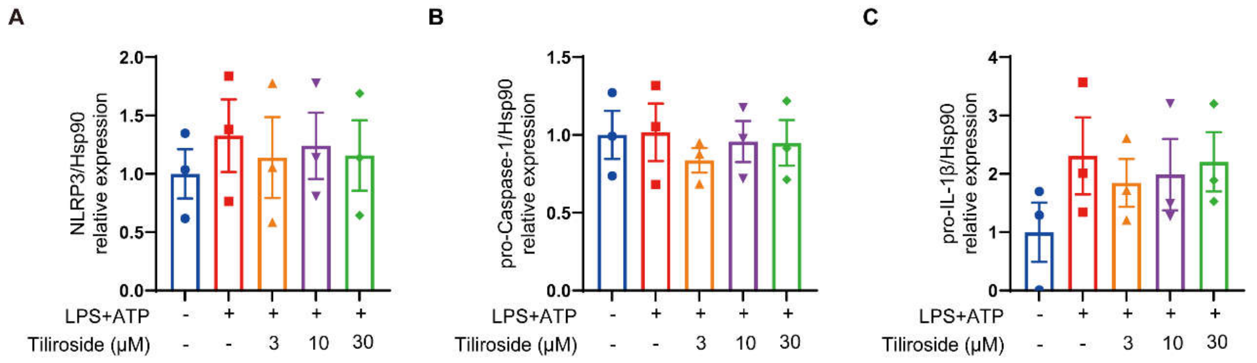


**Figure S1. The effect of tiliroside on the expression of NLRP3, pro-Caspase-1, and pro-IL-1β in LPS plus ATP-induced THP-1 macrophages. (A-C)** THP-1 macrophages were pretreated with tiliroside or a vehicle for 24 h and then stimulated with LPS (2 μg/mL) for 5 h, followed by ATP (5 mM) treatment for 45 min. NLRP3 **(A)**, pro-Caspase-1 **(B),** and pro-IL-1β **(C)** expression were determined by Western blotting, and the relative expression levels were quantified (n=3). Data are mean ± SEM. These data are related to Figure 1C.

**Supplementary Figure S2**

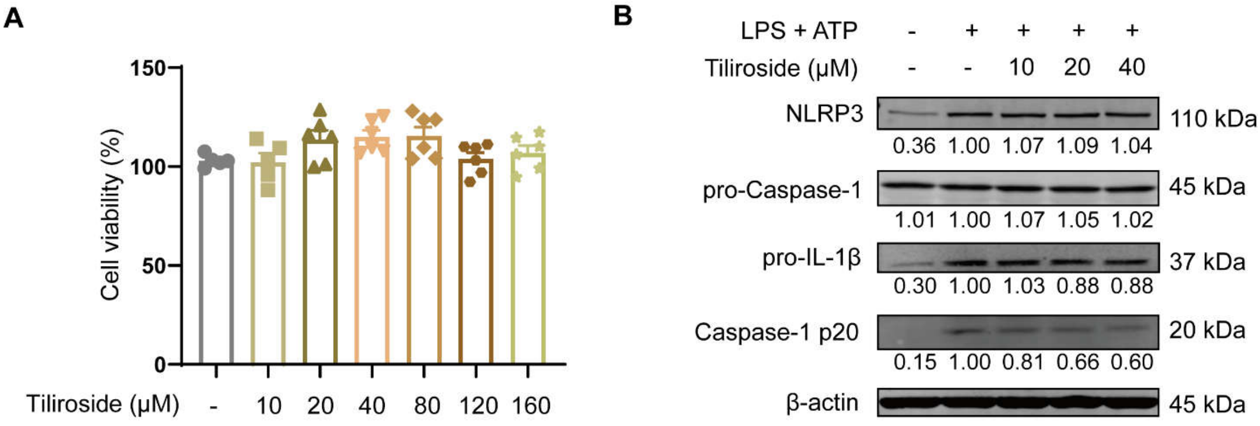


**Figure S2. Tiliroside inhibits NLRP3 inflammasome activation in BMDMs. (A)** Cell viability was measured by CCK-8 assay using BMDMs treated with different concentrations of tiliroside for 30 h. **(B)** BMDMs were pretreated with tiliroside or a vehicle for 24 h and then stimulated with LPS (100 ng/ml) for 5 h, followed by ATP (5 mM) treatment for 30 min. Protein levels of Caspase-1 p20, NLRP3, pro-Caspase-1, and pro-IL-1β in cell lysates were determined by Western blotting.

**Supplementary Figure S3**

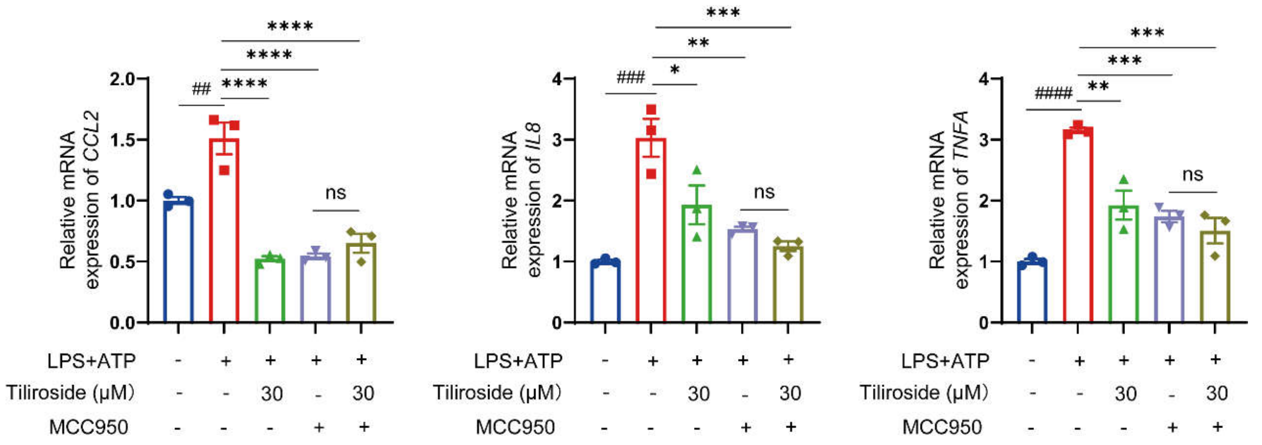


**Figure S3. The effect of tiliroside on the expression of pro-inflammatory mediators can be blocked by NLRP3 inhibitor MCC950.** THP-1 macrophages were pretreated with tiliroside (30 μM) for 23 h, followed by co-treatment with MCC950 (0.01 μM) for an additional 1 h, and then stimulated with LPS (2 μg/ml) for 5 h, followed by ATP (5 mM) treatment for 45 min. CCL2, IL-8, and TNFA gene expression levels were analyzed by qRT-PCR (n=3). Data are mean ± SEM. $^{##}P < 0.01$, $^{###}P < 0.001$, $^{####}P < 0.0001$, $^{*}P < 0.05$, $^{**}P < 0.01$, $^{***}P < 0.001$, $^{****}P < 0.0001$.

**Supplementary Figure S4**

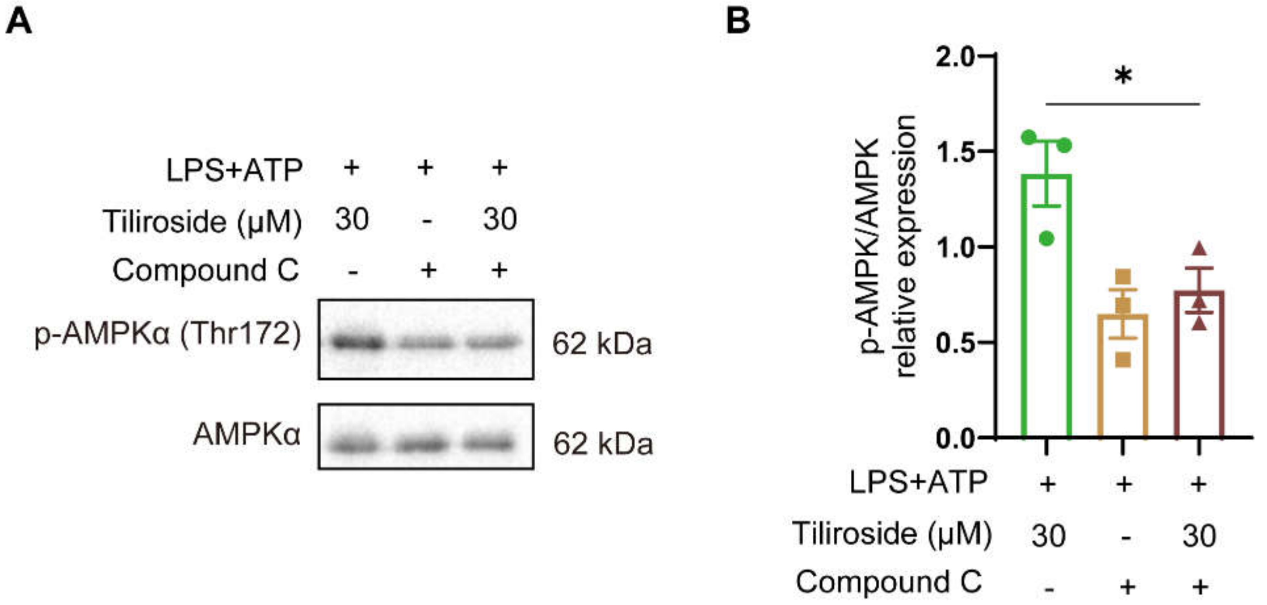


**Figure S4. Compound C blocks AMPK activation in THP-1 macrophages.** THP-1 macrophages were pretreated with tiliroside (30 μM) for 22 h, followed by co-treatment with Compound C for an additional 2 h, and then stimulated with LPS (2 μg/ml) for 5 h, followed by ATP (5 mM) treatment for 45 min. **(A, B)** Protein levels of p-AMPKα (Thr172) and AMPKα were determined by Western blotting **(A)** and quantification of p-AMPKα (Thr172) normalized to total AMPKα (n=3) **(B)**. Data are mean ± SEM. *$P < 0.05$.

**Supplementary Figure S5**

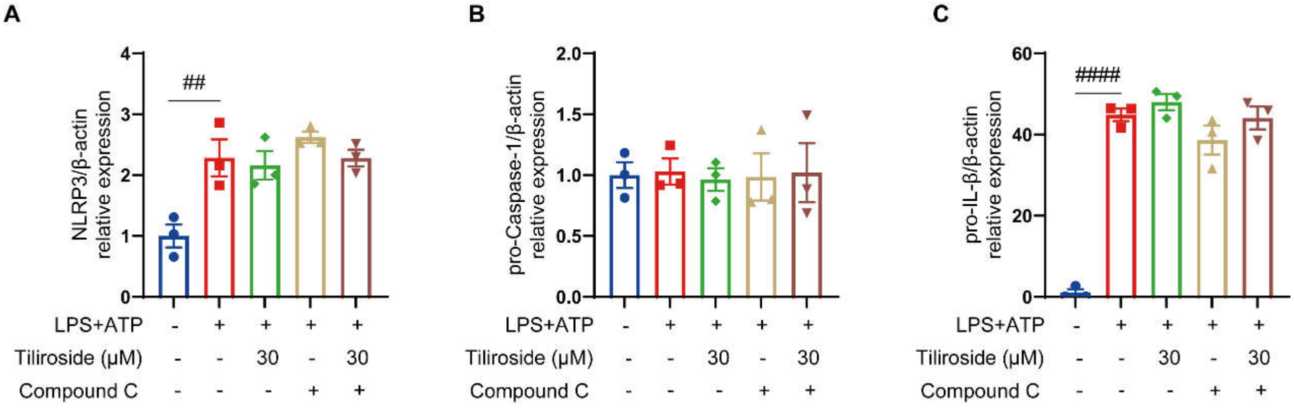


**Figure S5. The effect of Compound C on tiliroside-mediated regulation of NLRP3, pro-Caspase-1, and pro-IL-1β in LPS plus ATP-induced THP-1 macrophages. (A-C)** THP-1 macrophages were pretreated with tiliroside (30 μM) for 22 h, followed by co-treatment with Compound C for an additional 2 h, and then stimulated with LPS (2 μg/mL) for 5 h, followed by ATP (5 mM) treatment for 45 min. NLRP3 **(A)**, pro-Caspase-1 **(B),** and pro-IL-1β **(C)** were determined by Western blotting, and the relative expression levels were quantified (n=3). Data are mean ± SEM. $^{\#\#}P < 0.01$, $^{\#\#\#\#}P < 0.0001$. These data are related to Figure 3A.
